# Ophthalmic Care at an Academic Medical Centre for Patients who were Incarcerated or in Immigration Detention

**DOI:** 10.21203/rs.3.rs-6050144/v1

**Published:** 2025-04-21

**Authors:** Nathan Scott, Lauren Wedekind, Jimmy Chen, Timothy Sestak, Logan Sigua, Jazmyn Yap, Sidney Lin, Fritz Gerald Kalaw, Kyle Marra, Jeffrey Lee

**Affiliations:** Shiley Eye Institute, University of California San Diego; University of California San Diego; Shiley Eye Institute, University of California San Diego; University of California San Diego

**Keywords:** Incarceration, Immigration, Detention, Inequities, Follow-up, Jail, Prison, Diversity, equity, inclusion

## Abstract

**Background/Objectives:**

Ophthalmic care for patients who are incarcerated or in immigration detention presents complex challenges for patients and providers. There remains a paucity of literature investigating ophthalmic care for these marginalised populations. We investigated delivery of ophthalmic care in an academic health system to patients who were incarcerated or in immigration detention.

**Subjects/Methods:**

We conducted a retrospective observational study and secondary analysis of electronic health record data for inpatient, outpatient, and surgical visits between 1 November 2018 and 30 September 2023 in an ophthalmology department at an academic medical centre in the United States for patients who were incarcerated or in immigration detention. Attendance at outpatient clinic visits and follow-up based on ophthalmic subspecialty, loss to follow-up, delays in follow-up, rates of ancillary testing were analysed.

**Results:**

1628 visits were extracted for 401 patients, for which 1084 were attended [2.7 ± 3.6 per patient] and 544 were canceled or no-showed [1.4 ± 1.8 per patient]. Patients attended 100 [68.5%] of cornea, 116 [64.4%] of glaucoma, 2 [28.6%] of neuro-ophthalmology, 139 [58.2%] oculoplastics, 9 [36.0%] of paediatrics/strabismus, and 109 [54.5%] of retina visits. Using a 2-week grace period for follow-up, 72 patients (40.9% with complete follow-up data) completed follow-up within 2 weeks. 229 patients (57.1%) were lost to follow-up at the end of the study period.

**Conclusions:**

Delays in care and visit attrition were substantial for patients who were incarcerated or in immigration detention. More work is needed to quantify outcomes and address disparities in care for these marginalised communities.

## INTRODUCTION

In the United States (US), approximately 2 million people are incarcerated before or after trial in 100 federal prisons, 1600 state prisons, 3000 local jails, 140 immigration detention centres, and other facilities.^1^ Of these, approximately 18,000 are held criminally and civilly for immigration-related reasons by the U.S. Marshals Service.^1^ Separately, 38,000 people in the US are civilly detained by US Immigration and Customs Enforcement in federal or privately run immigration detention facilities because they are asylum seekers and/or facing deportation, rather than for any suspected crime.^1^

While correctional facilities and detention centres in the US are required to provide healthcare to people who are incarcerated or detained,^2^ studies have reported substantial disparities in health outcomes and health care access for these marginalised populations.^3–5^ Many facilities are remote and unable to provide onsite primary and subspecialty care, and thus partner with external health care systems to deliver care to patients.^6^ In these cases, coordination of transportation, security, and initial and follow-up appointments for patients is required for patients to receive necessary care. A recent systematic review of studies from 16 distinct cohorts found that surgical complications, delayed hospital presentations, and complex cases were more prevalent in incarcerated versus non-incarcerated individuals.^7^

Ophthalmic care for patients who are incarcerated or in immigration detention also presents complex challenges to patients and providers. Ocular and orbital trauma,^8,9^ as well as chronic ophthalmic conditions that may be manifestations of or affected by systemic conditions (e.g., diabetic retinopathy and glaucoma)^10,11^ require regular follow-up and may require surgical management. A study of follow-up care in an outpatient ophthalmology setting at a single academic medical centre for patients who were incarcerated found that over 60% of patients were lost to follow-up; of those who were not lost to follow-up, over 40% experienced delays in care.^12^ There remains a paucity of literature investigating health care delivery across ophthalmic subspecialties for these marginalised populations. In particular, data on ophthalmic care provision for patients who are in immigration detention are especially sparse. To address this gap in knowledge, we investigated the delivery of inpatient, outpatient, and surgical ophthalmic care to these populations in an academic health system.

## METHODS

The UCSD Institutional Review Board approved this study (IRB #120516) and the described research adheres to the tenets of the Declaration of Helsinki and Health Insurance Portability and Accountability Act (HIPAA) of 1996. The study’s participant selection, methodology, results, and discussion adheres to the Strengthening the Reporting of Observational Studies in Epidemiology (STROBE) guidelines. Informed consent was waived by the UCSD IRB in this retrospective study.

### Data source and collection

We conducted a retrospective observational study using data from the electronic health record system (Epic, Verona, WI, USA) at a single academic medical centre. The study population included: 1) patients who were incarcerated at local jails or state or federal prisons, or detained at immigration detention facilities at the time of the study, and 2) patients had one or more in-office visits with the ophthalmology service at University of California San Diego (UCSD) from 1 November 2018 to 30 September 2023, inclusive. We employed the Epic SlicerDicer tool (Epic Systems, Verona, WI, USA) to query this population. Between 19 October 2023 and 25 May 2023, we extracted data for study participants’ visits within the study period.

We manually extracted data from ophthalmology encounters in the emergency department (ED), and hospitalisation follow-ups, outpatient clinic visits, and surgeries for patients who were incarcerated. Demographic and visit data were extracted for each patient’s visit. Demographic data included: date of birth, gender, ethnicity, race, and insurance provider (e.g., County Medical Services, State of California/State Prison, Immigration Health Services, US Marshal Services, etc.). Visit data included: type of visit (i.e., outpatient in-person, telemedicine, ED, hospitalisation follow-up, or surgical); subspecialty of provider (i.e., comprehensive ophthalmology, glaucoma, neuro-ophthalmology, oculoplastics, paediatric ophthalmology/strabismus, retina), visit attendance (attended vs. canceled or no-showed), the first three 3 ophthalmic diagnoses that were addressed in the visit, visual acuity (VA), intraocular pressure (IOP), interventions planned and performed, ancillary testing received and planned (optical coherence tomography [OCT], Humphrey visual fields [HVF], intraocular lens calculations such as optical biometry or immersion scans for cataract surgery lens calculations, corneal topography [Pentacam], fluorescein or indocyanine angiography, and fundus photography), recommended time to follow-up, and recommended time to planned interventions. Planned interventions which were performed included laser procedures (capsulotomy, laser peripheral iridotomy, selective laser trabeculoplasty) and surgical procedures (cataract surgery, vitrectomy, orbital wall fracture repair, etc). Intravitreal injections were excluded as the decision for injection for the majority of patients in this cohort was contingent on their visit imaging (i.e. diabetic macular oedema).

**Figure 1:**
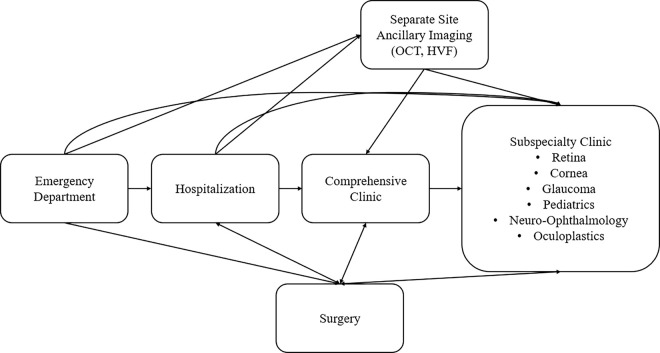
Flowchart of Visits for Patients who are Incarcerated at the University of California at San Diego. Each block represents a distinct setting/location where a visit occurs. At our institution, ancillary testing (i.e. Humphrey visual fields [HVF], lens calculations, and optical coherence tomography [OCT] among other tests) occur at a different location than the comprehensive and subspecialty clinics.

Patients who were incarcerated presented to UCSD either via the ED or referral to a comprehensive or subspecialty clinic. Patients who presented to the ED and required ophthalmic consultation were either admitted and followed by an ophthalmologist or discharged with follow-up to a clinic specific for patients who were incarcerated. If ancillary imaging such as OCT or HVF was required, patients were scheduled to present at a separate imaging appointment at a different location before their clinical visit as imaging equipment was not available at the office for which the patient visit occurred. Each subspecialty was available at least 1 day every week. Surgery could be arranged for inmates in either the inpatient or outpatient setting in the hospital operating rooms. A flowchart of hospital, clinic, imaging, and surgery visits for patients who are incarcerated and present to care at UCSD is shown in Fig. 1.

### Statistical Analysis

All data analysis was performed using R version 4.3.2 (Vienna, Austria).^13^ The total number of patients who met inclusion criteria was enumerated. Mean and standard deviations were calculated for counts of each category of demographic data. The most common ophthalmic diagnosis were tabulated. Visits were categorised into the following: emergency department, hospitalisation follow-up (i.e. seen by ophthalmology during inpatient admission), outpatient in-person (i.e. clinic visit), outpatient telemedicine, or surgical. Visit attendance, categorised as attended vs. canceled or no-show, was enumerated for each visit category. Canceled and no-show visits were grouped together because ancillary staff often opened visit encounters before the patient arrived, and cancellations or no-shows were often marked after the visit was over. Visit attendance by sum and percentage was calculated for each visit type, on a per patient basis, and also by subspecialty. Ancillary tests were tabulated by the test performed. Due to the non-normal distribution of visit attendance, the Mann-Whitney U test was used to assess statistical differences between visit attendances by subspecialty, with p ≤ 0.05 thresholded for statistical significance.

Follow-up intervals were calculated between each visit. Delays in follow-up were calculated as the difference between the recommended time interval for follow-up and the actual follow-up interval with a 2-week grace period. We also ascertained loss to follow-up, which was defined as a lack of follow-up visits with ophthalmologists in this health system after incarceration or after the end of the study period.

The best corrected distance VA was reported per eye for each visit was converted from Snellen to logMAR. The mean and standard deviation for visual acuity recorded in the first visit, as well as for the last visit, were calculated, for all patients and for various subsets of patients, based on ophthalmic diagnoses that were reported during the study period: retinal detachments, cataracts, glaucoma, or orbital fractures. These calculations were also calculated for IOP for the first visit and last visit. Due to the non-normal distribution of these values, the Mann-Whitney U test was used to assess statistical differences, with p ≤ 0.05 thresholded for statistical significance.

## RESULTS

### Patient Characteristics

Overall, 401 patients were included in this study. The mean age at presentation was 44.2 ± 14.2 years. The majority of patients were male (n = 363 [90.5%]) and reported an ethnicity of Non-Hispanic or Latino (n = 189 [47.1%]). The majority of patients did not report their race (n = 209 [52.1%]), although the most common reported races were White (n = 117 [29.2%]) and Black (n = 62 [15.4%]). The most common insurance coverage for these patients were county medical services (n = 191 [47.6%]) and California state prison (n = 134 [33.4%]). The average distance from facility to clinic was 44.8 ± 55.2 miles. Complete demographic data is shown in Table 1.

### Visit Statistics

A total of 1628 visits were extracted for the 401 included patients, for which 1084 visits were attended [2.7 ± 3.6 per patient] and 544 visits were canceled or no-showed [1.4 ± 1.8 per patient]. Of the 1413 outpatient visits, 1409 were in-person and 4 were telemedicine visits. Patients attended 874 outpatient in-person visits [2.2 ± 2.9 per patient], representing 53.1% of all visits or 62.1% of outpatient in-person visits. Patients also no-showed or cancelled, 535 outpatient in-person visits [1.3 ± 1.8 per patient], or 32.5% of all visits or 37.9% of outpatient in-person visits (the study no-show rate). In our study, there were also 93 ED visits [0.2 ± 0.5 per patient] and 64 hospitalisation follow-up visits [0.2 ± 1.0 per patient]. Patients overall attended 2 telemedicine visits [0.0 ± 0.1 per patient] and canceled or no-showed 2 telemedicine visits [0.0 ± 0.1 per patient]. Patients were scheduled for 58 surgeries overall, for which patients attended 51 surgeries [0.1 ± 0.4 per patient] and canceled or no-showed 7 surgeries [0.0 ± 0.1 per patient. The most common procedures performed were vitrectomies (n = 19), cataract surgery (n = 12), and glaucoma drainage devices (n = 5). These data are shown in **Supplementary Table 1**. Visit attendance data are summarised in Table 2. Ancillary tests (i.e. OCTs, HVFs) were requested in follow-up for 131 attended outpatient visits (14.9% of attended visits), although ancillary tests were only performed in follow-up for 22 attended outpatient visits (16.8% of visits where ancillary tests were requested) [data not shown].

Of the 874 attended outpatient visits and 535 canceled/no-showed outpatient visits, 401 [65.0%] visits were attended and 216 [35.0%] were canceled/no-showed in the comprehensive clinic. Patients attended 100 [68.5%] of cornea, 116 [64.4%] of glaucoma, 2 [28.6%] of neuro-ophthalmology, 139 [58.2%] ofoculoplastics, and 9 [36.0%] of paediatrics/strabismus, and 109 [54.5%] of retina visits. Patients generally canceled or no-showed to neuro-ophthalmology,oculoplastics, paediatrics/strabismus, and retina visits more often compared to the comprehensive clinic (p < 0.05). This data is shown in Table 3.

**Figure 2:**
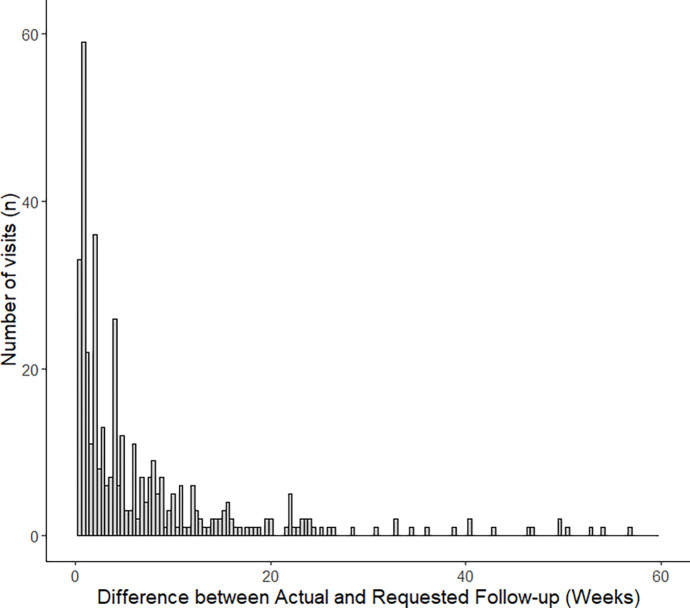
Differences in Actual and Requested Follow-up, or Delays in Follow-up.

Complete follow-up data with attended follow-ups and return to clinic requests from the prior visit were available for 473 attended visits from 176 patients.Overall, the mean difference between actual and requested follow-up, or delay in follow-up, was 9.1 ± 19.4 weeks. Using a 2-week grace period for follow-up, 72 patients (40.9% with complete follow-up data) completed follow-up within 2 weeks. The distribution of delays in follow-ups is shown in Fig. 2. Of the 401patients included in the study, 229 patients (57.1%) were lost to follow-up at the end of the study period. We were not able to accurately calculate delays infollow-up ophthalmic care for patients seen for ophthalmology consult services in the emergency department, as some of these data were incomplete ormissing.

### Visual Acuity and Intraocular Pressure Trends

The mean visual acuity (logMAR) at first and last visit recorded in the study interval was 0.5 ± 0.7 and 0.4 ± 0.7 respectively (p = 0.16). The mean IOP at the first and last visit recorded in the study interval was 14.6 ± 5.0 and 14.4 ± 5.0 mmHg respectively (p = 0.35). Subgroup analyses of VA and IOP by the most common diagnoses (retinal detachment, cataract, orbital wall fracture, and glaucoma) did not yield any statistically significant differences (**Supplementary Fig. 2)**.

## DISCUSSION

This study used EHR data to quantify visit attendance and adherence as well as delays in follow-up care and loss to follow-up (LTFU) experienced by patients in an academic health system who were incarcerated or in immigration detention. Our study has two key findings: 1) visit adherence and attrition are substantial issues for patients who are incarcerated, and when follow-up occurs, it tends to occur at longer than recommended intervals; and 2) utilisation and attendance of ancillary testing was also low for this population.

First, visit adherence and attrition were found to be substantial issues for patients who are incarcerated or in immigration detention. LTFU and follow-up delay rates (using a 2-week grace period for follow-up) were 57.1% and 40.9% (Fig. 2), respectively. LTFU for comprehensive ophthalmology visits in the present study was consistent with data from previous reports on ophthalmic care delivery for patients who were incarcerated.^12,14^ On subgroup analyses within the present study, paediatric ophthalmology had a relatively high incidence of visit non-adherence, consistent with reports of low visit adherence among the general population.^15,16^ By contrast, the present study population had a substantially higher rate of visit cancellation or no-show for oculoplastics clinic appointments than the general population in the literature.^17^ Previous studies have shown that delays in follow-up care for patients with chronic conditions that require long-term follow-up and management (e.g., glaucoma) can substantially worsen outcomes.^18^ In diseases in which vision is not usually immediately threatened (i.e. orbital wall fractures without entrapment and glaucoma), patients may not be aware of the need for routine follow-up to prevent complications of their diseases or vision loss. Prior work has demonstrated low rates of health literacy among patients who are incarcerated, and suggested that interventions to increase health literacy may improve follow-up rates.^19^

Factors cited from previous studies for non-adherence and attrition in health care for the general population include socioeconomic disparities, long waiting times, and transportation to health care facilities.^14,20–22^ Logistical and communication difficulties in scheduling and transporting patients to appointments pose substantial barriers in access to care for patients who are incarcerated or in immigration detention. Many jails, prisons, and detention facilities have policies that do not allow for disclosing specific follow-up details of inmates for security reasons.^10^ Also, nearly half of patients had coverage through County Medical Services, reflective of their incarceration in city jails. This subpopulation may experience higher rates of turnover given that jails hold individuals prior to trials and/or relocation to prisons or release to the community. Administrative staff of jails, prisons, and detention facilities must therefore coordinate future appointments directly with staff at health care facilities, which can delay and complicate timely health care delivery.^10^ Individuals who are incarcerated have high levels of distrust in the health care system, which could also contribute to attrition and LTFU.^23^ Other marginalised populations (e.g., uninsured, undocumented, and previously incarcerated people) have also been shown to have low rates of follow-up.^3,24^ Randomised trials within the general population have reported improvements in follow-up by implementing text message and telephone reminders for appointments^25^ and employing patient navigators and social workers to engage with patients to provide appropriate health care and social resources.^26^

Further work is needed to understand how we can improve access to ophthalmic care and follow-up for patients who are incarcerated or detained.

Second, there were particularly low rates of attendance and utilisation of ancillary testing. Ancillary testing, including imaging (e.g., OCT and ultra-widefield fundus imaging) and visual fields, provides essential information on the management of various ophthalmic conditions. For example, RNFL OCTs and HVF are ideally used to assess management of primary open angle glaucoma, and macula OCTs are essential in the management of diabetic retinopathy and retinal detachments. Ancillary tests were requested in follow-up for 15% of attended outpatient visits; however, these tests were only completed in follow-up for 17% of those respective visits (data not shown). The health system in which this study took place requires ancillary testing to be completed at separate appointments following the visits at which tests are requested, which may logistically limit visit adherence, as two separate instances of transportation would be required for a given visit. Low rates of requesting ancillary testing could reflect biases by providers: providers may be under-ordering tests for patients who are incarcerated or detained. This disparity may be due to expectations of barriers to adherence and attendance, as well as stigma and bias against this marginalised population.^27^

To improve the utilisation of ancillary testing, interdisciplinary teams could combine the provision of ancillary testing and outpatient clinic visits, such that patients could see their ophthalmologist and complete ancillary testing on the same date. Future research could integrate greater sample sizes of patients who are incarcerated who are able to complete ancillary testing. These measures are necessary as our study found no statistical differences between measurements of VA and IOP at patients’ first vs. last visits. It is likely that VA and IOP broadly are inadequate measures of patient outcomes alone. The lack of statistically significant differences persisted even within sub-analysis of patients with retinal detachments, cataracts, and orbital wall fractures; however, these data were limited by small sample sizes. Future work should focus on analysing objective metrics for ophthalmic disease severity to better understand outcomes of patients who are incarcerated with ophthalmic disease.

This study has multiple limitations. First, our study was limited in sample size. This may limit the generalisability of our findings to other incarcerated populations domestically and internationally. Second, we did not compare follow up rates and patient outcome metrics to the general population at UCSD. Although this broader analysis was out of the scope of our study, future work may focus on comparing visit adherence and patient outcomes between these populations. Third, we did not analyse medication adherence as we found medication documentation and review was often incomplete, consistent with prior literature.^28^ Future studies and interventions should focus on analysing how patients who are incarcerated use medications and how to improve medication adherence.

## Conclusion

The present study is among the first to investigate delivery of ophthalmic care to patients who are incarcerated or detained. We demonstrate that visit attrition is particularly high in this marginalised population, which in turn limits our ability to objectively study the outcomes of these patients. Multidisciplinary collaborations between clinicians, ancillary staff, hospital administrators, jail/detention staff, and social workers, will be necessary to improve healthcare delivery, including improved visit adherence and outcomes, for these patients.

## Figures and Tables

**Table 1:** Demographic and clinical characteristics of patients who were incarcerated or in immigration detention.

Demographic	Count
**Total Number of Patients** (n)	401
**Age at Presentation** (Mean ± Standard Deviation Years)	44.2 ± 14.2
**Average Distance from Facility to Clinic** (Mean ± Standard Deviation Miles)	44.8 ± 55.2
**Gender** (n [%])	
Female	38 [9.5%]
Male	363 [90.5%]
**Ethnicity** (n [%])	
Hispanic or Latino	130 [32.4%]
Non-Hispanic or Latino	189 [47.1%]
Unknown	82 [20.5%]
**Race** (n [%])	
White	117 [29.2%]
Black	62 [15.4%]
Hawaiian or Pacific Islander	1 [0.3%,]
American Indian or Alaska Native	3 [0.8%,]
Asian	9 [2.2%,]
Other/Unknown	209 [52.1%]
**Insurance Status** (n [%])	
County Medical Services	191 [47.6%]
State of California / State Prison	134 [33.4%]
Immigration Health Services	35 [8.7%,]
US Marshal Services	21 [5.2%]
Detention	6 [1.5%]
Medi-Cal	6 [1.5%]
Federal Prison	3 [0.8%]
Commercial	1 [0.3%]
Unknown	4 [1.0%]
**Most Common Primary Ophthalmic Diagnoses at Presentation** (n)	
Orbital Wall Fracture	64
Open or Closed Angle Glaucoma	39
Cataracts	28
Vitreous Floaters or Posterior Vitreous Detachment	14
Retinal Detachment	14
Dry Eyes	11
Blurry Vision	10
Corneal Scar	10
Pterygium	8
Corneal Epithelial Defect	7

**Table 2:** Overall and Per-patient Visit Attendance vs. Visit Cancellations and No-shows.

	Visit Attendance		
Visits	Attended	Cancellation or No-Show	Total (n)
Total Visits (n [%])	1084 [65.9%]	544 [34.1%]	1628 [100.0%]
Outpatient In-Person	874 [53.1%]	535 [32.5%]	1409 [86.6%]
Outpatient Telemedicine	2 [0.1%]	2 [0.4%]	4 [0.2%]
Emergency Department Visit	93 [5.7%]	N/A	93 [5.7%]
Hospitaliation Follow-Up	64 [3.9%]	N/A	64 [3.9%]
Surgical	51 [3.1%]	7 [0.1%]	58 [3.6%]
Per-Patient Total (Mean ± Standard Deviation)	2.7 ± 3.6	1.4 ± 1.8	4.1 ± 4.6
Outpatient In-Person	2.2 ± 2.9	1.3 ± 1.8	3.5 ± 4.0
Outpatient Telemedicine	0.0 ± 0.1	0.0 ± 0.1	0.0 ± 0.1
Emergency Department Visit	0.2 ± 0.5	N/A	0.2 ± 0.5
Hospitaliation Follow-Up	0.2 ± 1.0	N/A	0.2 ± 1.0
Surgical	0.1 ± 0.4	0.0 ± 0.1	0.1 ± 0.5

Percentages were calculated as a proportion of the total number of visits.

**Table 3:** Outpatient Visit Attendance by Subspecialty.

	Visit Attendance		
Subspecialty	Attended (n [%])	Cancellation or No-Show (n [%])	Total (n)
Comprehensive	401 [65.0%]	216 [35.0%]	617
Cornea	100 [68.5%]	46 [31.5%]	146
Glaucoma	116 [64.4%]]	64 [35.6%]	180
Neuro-Ophthalmology	2 [28.6%]	5 [71.4%] *	7
Oculoplastics	139 [58.2%]	100 [41.8%] *	239
Paediatrics/Strabismus	9 [36.0%]	16 [64.0%] *	25
Retina	109 [54.5%]	90 [45.5%] *	199

* p < 0.05

Percentages were calculated out of the total visits available for each subspecialties. P-values comparing the distribution of attended and canceled visits were calculated comparing each subspecialty to the comprehensive clinic.
